# Exploring the Potential Link Between Autoimmune Diseases and Pan-Cancer: A Multidatabase Mendelian Randomization Analysis

**DOI:** 10.1155/jimr/6468979

**Published:** 2025-04-27

**Authors:** Chenguang Wang, Zhiyong Liu, Yuhao Zhou, Yan He, Yashu Zhang, Shiqi Chen, Wenqing Yang, Lijun Fan

**Affiliations:** Centre for Endemic Disease Control, Chinese Centre for Disease Control and Prevention, Harbin Medical University, Harbin City 150081, Heilongjiang Province, China

**Keywords:** AID, autoimmune disease, cancer, malignancy, UK Biobank

## Abstract

**Background:** The relationship between autoimmune diseases (AIDs) and cancer is unclear and this study aimed to investigate the relationship between AIDs and cancer at the genetic level using Mendelian randomization (MR).

**Methods:** The study employed two-sample MR and meta-analysis to investigate the association between AIDs and 33 types of cancer, following STROBE-MR guidelines. Single nucleotide polymorphisms (SNPs) associated with AIDs were used as instrumental variables, with data from FinnGen, UK Biobank, and other databases. MR analyses included sensitivity checks, heterogeneity assessments, and reverse causality tests, using multiple MR methods (inverse-variance weighted (IVW), weighted median, MR-Egger, etc.). Meta-analysis was performed on validated results to confirm findings, with statistical analyses conducted using R software.

**Results:** The results identified eight significant associations in both discovery and replication stages. Key findings include that myasthenia gravis (MG) significantly increases the risk of oral cavity cancer, multiple sclerosis (MS) is linked to increased risks of chronic lymphocytic leukemia (CLL) and small intestine cancer, and ulcerative colitis (UC) has mixed effects, reducing the risk of uterine cervix and larynx cancers, but increasing risks for pancreatic and bladder cancers. Meta-analysis confirmed eight secondary findings, highlighting pathogenic associations such as type 1 diabetes with esophagus cancer and protective effects like systemic lupus erythematosus (SLE) against acute myelocytic leukemia.

**Conclusions:** This study provides evidence of a causal relationship between multiple AIDs and different cancer risks at the genetic level and provides a reference for the health management of patients with AIDs.

## 1. Introduction

Cancer is a disease characterized by the uncontrolled growth and division of cells. The development of cancer involves genetic mutations, abnormalities in gene expression regulation, and environmental factors, though the precise causes and mechanisms are not yet fully understood. Autoimmune diseases (AIDs) represent a class of disorders where the immune system fails to maintain tolerance to self-antigens, leading to attacks on the body's own tissues and cells, resulting in chronic inflammation and organ damage [1, 2]. Common AIDs include systemic lupus erythematosus (SLE), multiple sclerosis (MS), Crohn's disease (CD), ulcerative colitis (UC), myasthenia gravis (MG), rheumatoid arthritis (RA), and type 1 diabetes. The chronic inflammatory environment associated with AIDs may be linked to cancer development. Additionally, dysregulation of the immune system impairs immune surveillance, allowing abnormal cells to evade timely detection and elimination. Furthermore, certain treatments for AIDs, such as immunosuppressants, may also reduce immune surveillance, potentially contributing to cancer incidence.

Numerous studies have demonstrated a correlation between AIDs and cancer. Interestingly, different AIDs can either increase or decrease cancer risk. For instance, research indicates that RA is associated with an increased incidence of non-Hodgkin lymphoma (NHL) [3], while systemic sclerosis is linked to a higher risk of breast and lung cancers [4]. Primary Sjögren's syndrome is associated with a significantly increased overall cancer risk [5]. Conversely, some studies suggest that certain AIDs may be associated with a reduced risk of cancer; for example, RA has been linked to lower risks of gastric, rectal, and liver cancers [6] and SLE patients have shown a decreased incidence of breast and prostate cancers [7].

Mendelian Randomization (MR) can be used to assess the impact of exposures on disease risk [8], serving as a genetic epidemiology method to reveal potential causal relationships. MR addresses limitations inherent in traditional observational epidemiological studies, such as confounding factors and reverse causation [9]. By using single nucleotide polymorphisms (SNPs) as instrumental variables, MR allows for indirect examination of complex causal effects between exposures and diseases [10]. Additionally, the random distribution of SNPs meets the requirements for randomization in controlled trials, thus, enhancing the reliability of results [11].

The relationship between AIDs and cancer remains an area of ongoing research. This study aims to explore the associations between various AIDs and pan-cancer using MR methods, which will contribute to the development of personalized health management strategies for patients with different AIDs.

## 2. Methods and Materials

### 2.1. Study Design

**Figure 1:**
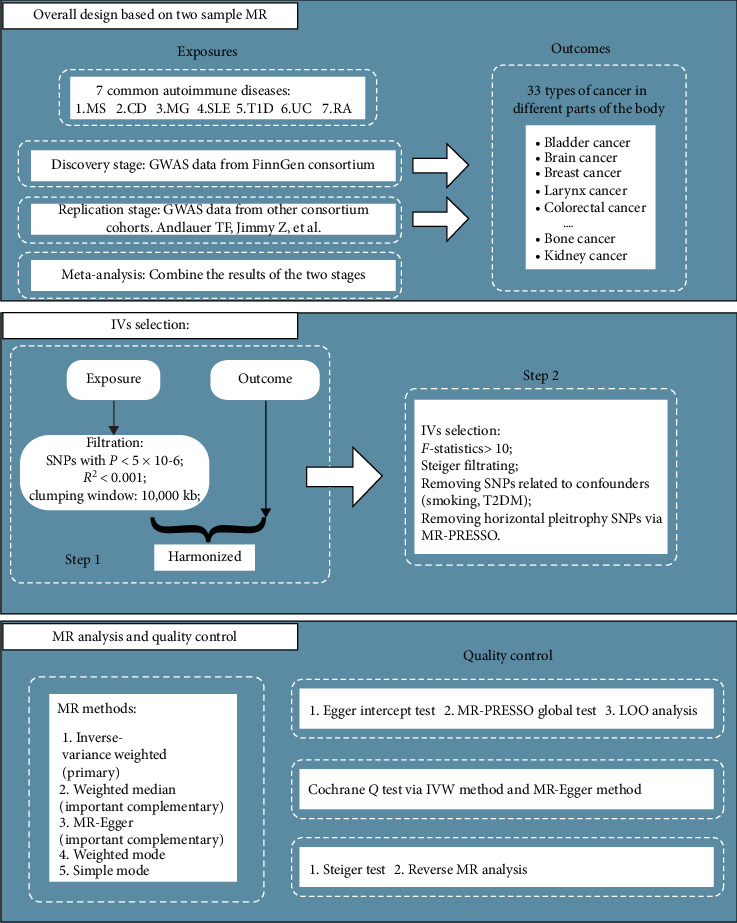
Study design flow diagram.

Our study is based on classic two-sample MR analyses [12–14] and meta-analysis theories, with the study design adhering to the quality control requirements outlined in the STROBE-MR guidelines [15]. A detailed flowchart of the study process is provided in Figure 1. We extracted SNPs strongly associated with AIDs as instrumental variables to investigate their relationship with 33 types of pan-cancer. To enhance the robustness of our results, we employed external database cross-validation, meta-analysis integration, and various MR cross-validation techniques.

The outcome data for the discovery stage and replication stage were obtained from the FinnGen database and a non-FinnGen database primarily comprising UK Biobank data, respectively. Each type of cancer was represented by data from at least two different GWAS databases. Results validated in both the discovery and replication stages were reported as primary outcomes. Additionally, we performed meta-analysis on results from both stages for the same cancer types, excluding those with pleiotropic effects. Positive results from the meta-analysis were reported as secondary outcomes. For quality control in individual MR analyses, we implemented a rigorous SNP extraction process, conducted multiple sensitivity analyses, heterogeneity assessments, and reverse causality checks to minimize potential biases (Figure 1).

### 2.2. Data Sources

#### 2.2.1. GWAS Data of AIDs

In the discovery stage, our seven exposure datasets (AIDs) were sourced from the FinnGen database [16]. The phenotypes included MS, CD, MG, SLE, type 1 diabetes, RA, and UC. FinnGen is a collaborative research project aimed at investigating the genomic and national health registry data of 500,000 Finnish individuals. It combines newly collected and legacy genotype data from the Finnish biobank with digital health records from the Finnish Health Registry (https://www.finngen.fi/en), offering new insights into disease genetics. As of August 2020 (the 5th version referenced in this paper), samples from 412,000 individuals have been collected, with analyses conducted on 224,737 individuals. FinnGen, with its unique Nordic healthcare system and demographic characteristics, provides a substantial resource for broad genetic discoveries.

In the replication stage, the seven exposure datasets (AIDs) were obtained from various databases. The MS phenotype data came from Andlauer TF's GWAS on MS susceptibility in German cohorts [17], which involved 15,283 Europeans, including 4888 cases of German ancestry and 10,395 German ancestry controls. CD phenotype data were derived from a study by Liu et al. [18] on 38 susceptibility loci for inflammatory bowel disease, encompassing 20,883 Europeans, with 5956 cases and 14,927 healthy controls. MG phenotype data were from a comprehensive GWAS by Chia et al. [19], including 1873 patients with acetylcholine receptor antibody-positive MG and 36,370 healthy individuals. This study also utilized expression data from skeletal muscle, whole blood, and tibial nerve in a transcriptome-wide association study (TWAS) to assess the impact of disease-associated polymorphisms on gene expression [19]. SLE phenotype data were provided by Glanville et al.'s [20] study on pleiotropy between depression and AIDs, identifying 28,479 cases of AIDs (across 14 traits) and 324,074 autoimmune controls. Type 1 diabetes phenotype data were sourced from Vincenzo Forgetta's study on rare genetic variants affecting type 1 diabetes risk, involving 9358 cases and 15,705 controls from 12 European cohorts, with 27 independent variants identified outside the MHC region [21]. UC phenotype data were provided by Ben Elsworth's European study, which included 2349 cases of UC and 460,494 healthy controls, with a total of 9,851,867 SNPs analyzed.

#### 2.2.2. GWAS Data of Multiple Cancers

The outcome measures in this study encompass 33 different types of cancer, including acute myeloid leukemia (AML), bladder cancer, bone cancer, brain cancer, breast cancer, bronchus and lung cancer, carcinoma of the gallbladder and extrahepatic biliary tract, chronic lymphocytic leukemia (CLL), chronic myeloid leukemia, esophageal cancer, eye and adnexa cancer, colorectal cancer, hodgkin lymphoma, intrahepatic bile duct carcinoma, kidney cancer, laryngeal cancer, liver cell carcinoma, malignant melanoma, malignant nonmelanoma, multiple myeloma, NHL, oral cavity cancer, oropharyngeal cancer, ovarian cancer, pancreatic cancer, prostate cancer, small intestine cancer, stomach cancer, testicular cancer, thyroid cancer, uterine cervix cancer, uterine corpus cancer, and vulvar cancer.

For the discovery stage, the 33 pan-cancer outcome data were primarily sourced from the UK Biobank database processed by the Pan-UKB initiative [22]. The UK Biobank is a prospective cohort study that collected in-depth genetic and phenotypic data from approximately 500,000 individuals aged 40–69 from across the United Kingdom. Cancer diagnoses in this dataset adhere to ICD-10 classification codes and we extracted relevant research data accordingly. Additionally, some cancer types were sourced through alternative channels. For instance, data on oral cavity cancer were extracted from Lessur C's contribution to the GWAS catalog (GCST012237), which includes publicly accessible data on 1223 cases and 2928 controls. Similarly, oropharyngeal cancer data were obtained from Lessur C's GWAS catalog contribution (GCST012237), involving 1090 cases and 2928 controls. Data for gallbladder cancer and extrahepatic bile duct carcinoma were sourced from Jiang's contribution to the GWAS catalog (GCST90041817), comprising 195 cases and 456,153 controls. Hepatocellular carcinoma and intrahepatic bile duct carcinoma data came from Jiang's contributions (GCST90043858 and GCST90043589) with 123 and 104 cases, respectively, and 456,225 and 456,244 controls. All UK Biobank cancer GWAS data used principal component analysis (PCA) to determine the main factors affecting incidence rates and employed multiple linear regression on the top 20 principal components to obtain accurate beta effect values for SNPs on the phenotypes.

#### 2.2.3. Instrumental Variables Selection

To obtain robust MR results, we implemented a rigorous process for selecting instrumental variables. We initially excluded SNPs with ambiguous or palindromic sequences, as well as those associated with confounding factors such as smoking and alcohol use, based on the GWAS Catalog database. We set the significance threshold for SNPs at 5e–6 and applied a 10,000 kb window.

We then reviewed minor allele frequencies (MAFs), filtering out SNPs with MAF less than 0.01 to eliminate rare variants. To address linkage disequilibrium, we calculated the independence of SNPs and excluded those with a correlation value below 0.001. We also calculated the *F*-statistic to identify and exclude weak instrumental variables, discarding SNPs with an *F*-statistic below 10. Further statistical analysis revealed that the 2613 IV-related SNPs from the FinnGen database (Supporting Information 1: Table S1) had a median *F*-statistic of 35.114, with a range of 21.359–1772.760. Meanwhile, the 9154 IV-related SNPs from other databases, including UK Biobank (Supporting Information 1: Table S2), had a median *F*-statistic of 49.756, with a range of 25.363–1706.166. Detailed distributions of *F*-statistics are provided in Supporting Information 2: Table S1 and S2. These results suggest that the potential for weak instrument bias is minimal, aligning with our predefined quality control standards.

To further ensure the validity of our results, we utilized the MR-PRESSO method and Steiger filtering to address pleiotropy and reverse causation. Finally, we retained exposure phenotypes and their associated instrumental variables if more than three IVs remained for subsequent analysis.

#### 2.2.4. MR Analysis

Our study employed five different MR methods: inverse-variance weighted (IVW), weighted median [23], MR-Egger [24], weighted mode, and simple mode. Among these, the IVW method is considered the most robust. It assumes no pleiotropy among instrumental variables and provides stable and accurate causal estimates by combining Wald estimates from each IV through meta-analysis. Thus, we use the IVW results as the primary indicator for evaluating our main findings, with the other four methods serving as supplementary.

The weighted median and MR-Egger methods both allow for pleiotropy among instrumental variables. The weighted median method permits up to half of the instrumental variables to exhibit pleiotropy, using a weighted approach to obtain results. In contrast, MR-Egger allows for pleiotropy among all instrumental variables, making it the most conservative of the five methods.

Weighted mode and simple mode are also significant supplementary methods. We consider the results most robust when the IVW *p*-value is less than 0.05 and the odds ratio (OR) directions are consistent across all five methods.

#### 2.2.5. Sensitivity, Heterogeneity, and Reserve MR

In our sensitivity analysis, we employed three methods: MR-Egger intercept, MR-PRESSO, and leave-one-out (LOO) analysis. The MR-Egger intercept method detects horizontal pleiotropy by examining the intercept term of the MR-Egger regression. If the intercept is significantly different from zero, it indicates the presence of horizontal pleiotropy. MR-PRESSO assesses global pleiotropy through a sampling-based approach, identifying whether pleiotropy affects the results across all instrumental variables [25]. The LOO method involves sequentially excluding each SNP to evaluate its impact on the outcome, with results displayed in a forest plot. If both the MR-Egger and MR-Egger intercept *p*-values are less than 0.05 and the LOO method does not reveal significant outliers, the risk of pleiotropy is considered low.

For heterogeneity analysis, we primarily used the IVW method and Cochran's *Q* test from MR-Egger, supplemented by funnel plots. A *p*-value greater than 0.05 in these tests indicates a low risk of heterogeneity. To detect reverse causation, we conducted a reverse MR analysis using the Steiger test for global assessment. In this reverse MR analysis, outcome data (pan-cancer) were treated as exposures and exposure data (AIDs) were treated as outcomes. This analysis followed the same procedures for extracting instrumental variables and data analysis as the forward MR. If the reverse MR results were not statistically significant and the Steiger test *p*-values were all below 0.05, the risk of reverse causation bias was considered low.

#### 2.2.6. Meta-Analysis and Statistics

We performed a meta-analysis of results from the discovery and replication stages that did not exhibit pleiotropy. The significance threshold for the meta-analysis was set at 0.01 and significant results from this meta-analysis were reported as secondary findings.

All MR analyses were conducted using R software (version 4.3.1). The extraction of instrumental variables, application of the five MR methods, sensitivity analyses, and result computations utilized the “TwoSampleMR” package (version 0.5.6) [26], the “MendelianRandomization” package (version 0.9.0), and the “MR-PRESSO” package (version 1.0). Meta-analysis and heterogeneity analyses were performed using the “meta” package and statistical plots were created using the “ggplot2” package.

## 3. Results

### 3.1. Instrument SNPs Filtration

Following the SNP extraction methods described, we achieved substantial results in both the discovery and replication stages. In the discovery stage, a total of 204 matched exposure–outcome pairs were identified. Specifically, CD was associated with 30 cancer outcomes, involving 174 unique SNPs with a median of 6 and an interquartile range (IQR) of 4–11. MS matched with 31 cancer outcomes, encompassing 249 unique SNPs with a median of 8 and an IQR of 5–12. MG was linked to 11 cancer outcomes, involving 50 unique SNPs, with a median of 5 and an IQR of 4–5. RA associated with 33 cancer outcomes, comprising 50 unique SNPs, with a median of 22 and an IQR of 12–34. SLE was matched with 32 cancer outcomes, involving 186 unique SNPs, with a median of 6 and an IQR of 4–11. Type I diabetes was associated with 33 cancer outcomes, including 530 unique SNPs, with a median of 16 and an IQR of 11–26. Last, UC matched with 33 cancer outcomes, involving 639 unique SNPs, with a median of 19 and an IQR of 12–31.

In the replication stage, we identified 200 matched exposure–outcome pairs. CD matched with 33 cancer outcomes, including 1072 unique SNPs, with a median of 32 and an IQR of 22–55. MS associated with 33 cancer outcomes, comprising 474 unique SNPs, with a median of 14 and an IQR of 9–27. MG linked to 31 cancer outcomes, involving 155 unique SNPs, with a median of 5 and an IQR of 4–9. RA was associated with six cancer outcomes, involving 29 unique SNPs, with a median of 5 and an IQR of 4–5. SLE matched with 33 cancer outcomes, including 199 unique SNPs, with a median of 6 and an IQR of 5–11. Type I diabetes was associated with 33 cancer outcomes, involving 6982 unique SNPs, with a median of 212 and an IQR of 166–228. UC matched with 32 cancer outcomes, comprising 243 unique SNPs, with a median of 8 and an IQR of 4–18.

All instrumental variables underwent thorough validation through MR-PRESSO and Steiger tests, confirming the exclusion of pleiotropy and reverse causation. We ensured that MAF exceeded 0.01, *R*^2^ was greater than 0.001, and *F* values surpassed 10, filtering out weak instruments while maintaining SNP independence. Additionally, SNPs with ambiguous or palindromic sequences and rare SNPs were excluded. Detailed results are available in Supporting Information 2: Table S1 and S2.

### 3.2. Main MR Results in Discovery Stage

**Figure 2:**
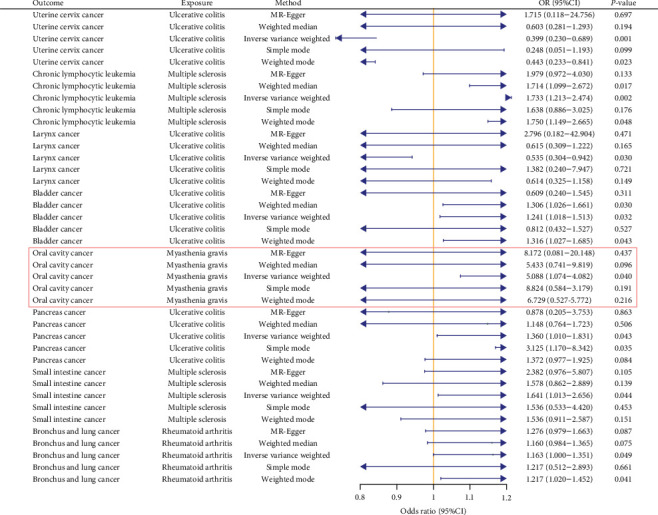
The positive results obtained in the discovery stage. The main results of the double database verification are shown in the red box.

In the discovery stage, we identified eight significant results. Notably, we observed that the UC phenotype was significantly associated with four types of cancer. Specifically, it exhibited a protective effect against uterine cervix cancer (*p*=0.001, OR = 0.399, 95% CI: 0.231–0.689) and larynx cancer (*p*=0.030, OR = 0.535, 95% CI: 0.304–0.942), while it was associated with an increased risk for pancreas cancer (*p*=0.043, OR = 1.360, 95% CI: 1.010–1.831) and bladder cancer (*p*=0.032, OR = 1.241, 95% CI: 1.018–1.513; Figure 2).

Additionally, our research revealed that the MS phenotype had a significant impact on two types of cancer: CLL (*p*=0.002, OR = 1.733, 95% CI: 1.214–2.474) and small intestine cancer (*p*=0.044, OR = 1.641, 95% CI: 1.014–2.656), both of which showed an exacerbating effect. We also found that the MG phenotype increased the risk of oral cavity cancer (*p*=0.04, OR = 5.088, 95% CI: 1.075–24.082) and the RA phenotype was associated with an increased risk of bronchus and lung cancer (*p*=0.049, OR = 1.163, 95% CI: 1.001–1.351). These principal positive findings were consistently supported by at least four of the five MR methods, including the IVW method. Detailed results are illustrated in Figure 2, with comprehensive analyses provided in Supporting Information 1.

### 3.3. Main MR Results in Replication Stage

In the replication stage, we identified eight significant results, one of which corroborated a finding from the discovery stage: MG was found to exacerbate the risk of oral cavity cancer (*p*=0.049, OR = 32.370, 95% CI: 7.230–143.552). Notably, the OR for this phenotype was higher than for other significant results, indicating a particularly pronounced exacerbating effect on oral cavity cancer.

**Figure 3:**
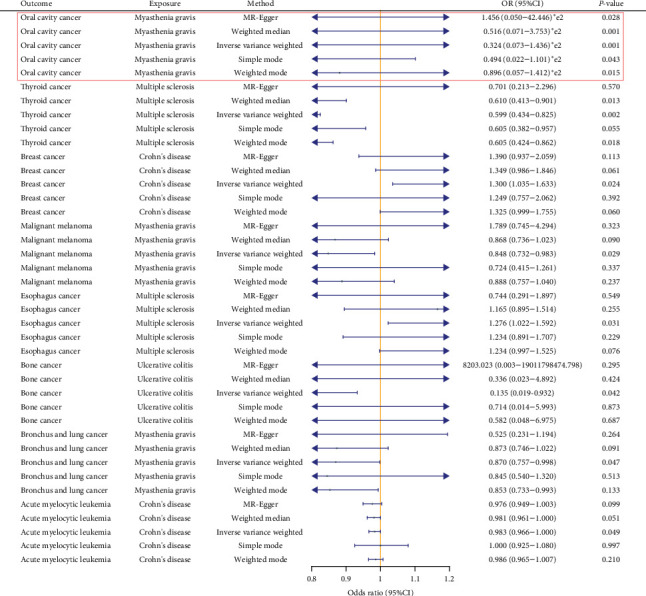
The positive results obtained in the replication stage. The main results of the double database verification are shown in the red box.

Additionally, we observed seven other significant associations. For the MS phenotype, we found two positive results: it was associated with a protective effect against thyroid cancer (*p*=0.002, OR = 0.599, 95% CI: 0.435–0.825) and an exacerbating effect on esophagus cancer (*p*=0.031, OR = 1.276, 95% CI: 1.023–1.592). For the CD phenotype, we identified two significant associations: it was associated with a protective effect against acute myelocytic leukemia (*p*=0.049, OR = 0.983, 95% CI: 0.967–0.999) and an exacerbating effect on breast cancer (*p*=0.024, OR = 1.300, 95% CI: 1.035–1.633). Similarly, the MG phenotype showed two positive results: it was associated with a protective effect against malignant melanoma (*p*=0.029, OR = 0.848, 95% CI: 0.732–0.983) and bronchus and lung cancer (*p*=0.047, OR = 0.870, 95% CI: 0.758–0.998). We also found that UC was associated with a reduced risk of bone cancer (*p*=0.042, OR = 0.135, 95% CI: 0.020–0.932). However, we excluded the result indicating that SLE exacerbates oral cavity cancer due to an anomalously high OR (243,681,410.561; Figure 3). To ensure the stability of our results, this outlier was removed from the analysis. Results from different MR methods are illustrated in Figure 3, with detailed analyses provided in Supporting Information 1.

### 3.4. Combined the MR Results of Discovery Stage and Replication Stage via Meta-Analyis

In summary, following validation through both the discovery and replication stages, our primary finding is that MG exacerbates oral cavity cancer. Subsequent meta-analysis combining all nonpleiotropic positive results from both stages revealed eight secondary findings. These include seven pathogenic associations: Type 1 diabetes increases the risk of esophagus cancer (random *p*=0.001, OR = 1.064, 95% CI: 1.024–1.106); MG exacerbates oral cavity cancer (random *p*=0.004, OR = 53.343, 95% CI: 3.509–810.950); MS increases the risk of small intestine cancer (random *p*=0.014, OR = 2.745, 95% CI: 1.231–11.425), uterine cervix cancer (random *p*=0.021, OR = 1.722, 95% CI: 1.085–2.735), and CLL (random *p*=0.046, OR = 1.771, 95% CI: 1.011–3.101); RA increases the risk of esophagus cancer (random *p*=0.021, OR = 1.722, 95% CI: 1.085–2.735); and SLE exacerbates eye and annexa cancers. Additionally, we identified one protective association: SLE has a protective effect against acute myelocytic leukemia (random *p*=0.040, OR = 0.775, 95% CI: 0.608–0.989). Detailed results are provided in Supporting Information 2 and 3.

### 3.5. Sensitivity, Heterogeneity, and Reverse MR

In assessing pleiotropy using the Egger intercept method from MR Egger, MR-PRESSO, and the LOO approach during the discovery stage, all significant results exhibited *p*-values greater than 0.05, and no substantial outliers were identified using the LOO method. This indicates a low risk of pleiotropy for the results from the discovery stage. Conversely, in the replication stage, we observed that the association between type 1 diabetes and bladder cancer had an MR-Egger intercept *p*-value of 0.013, which is below 0.05, suggesting the presence of pleiotropy. Consequently, this result was excluded from reporting and meta-analysis.

Regarding heterogeneity, Cochran's *Q* test using both the IVW method and MR-Egger method showed *p*-values greater than 0.05 in both the discovery and replication stages, indicating a low risk of heterogeneity. For reverse MR, results under the IVW method had *p*-values exceeding 0.05, suggesting statistical insignificance. Furthermore, Steiger test global *p*-values were well below 0.05, indicating a low risk of reverse causation bias in our results.

## 4. Discussion

Cancer remains a leading cause of death globally and presents a significant economic and social challenge [27]. AIDs, characterized by a loss of self-tolerance and subsequent immune-mediated destruction of the body's own tissues, occur in multiple organs or systems [28]. Immune dysregulation is believed to play a pathogenic role in the mechanisms of both AIDs and cancer and increasing evidence suggests a potential association between AIDs and malignancies [27]. Our study identifies several causal relationships between AIDs and cancer, providing new insights for clinical cancer screening and highlighting the need for increased vigilance among clinicians managing AID patients.

MG, an AID caused by antibodies that impair neuromuscular junction transmission, leads to muscle fatigue and weakness due to antigen cross-linking and complement activation [28–30]. The relationship between MG and cancer has been unclear, but our findings reveal a pathogenic causal effect of MG on oral cavity cancer (raw-*p*=0.040; OR = 5.088; 95% CI: 1.075–24.082), filling a gap in the literature and underscoring the importance of monitoring oral cavity health in MG patients.

In the realm of other AIDs, MS is a chronic demyelinating disease of the central nervous system, characterized by demyelinating lesions in the white and gray matter of the brain and spinal cord [31]. Research by Bahmanyar et al. [32] indicates that chronic inflammation induced by MS may weaken immune protection against cancer. Conversely, Fletcher et al. [33] suggest that MS might protect against cancer by upregulating immune activity, with Th1 and Th17 cells producing antitumor factors that help inhibit tumor cell proliferation. Previous studies have linked MS to various cancers. Wu et al. [34] found that AIDs are associated with an increased risk of small intestine cancer, potentially due to overactivation of IL-12 and IL-23 signaling, which leads to TH1 and TH17 immune responses and chronic inflammation. The IL-23/TH17/IL-17 axis might compromise local mucosal barriers in the skin, gut, and lungs, and suppress cytotoxic T cell-mediated antitumor immune surveillance [34]. A study involving natalizumab treatment for relapsing–remitting MS revealed rapid progression of low-grade cervical dysplasia to invasive cancer, with HPV being a major cause of cervical dysplasia and cancer. Although most women clear HPV infections within 2 years, immune system efficacy is crucial in virus clearance and immunosuppression is linked to increased risk of persistent HPV infection and cervical dysplasia [35]. The optimal cervical cancer screening interval for immunocompromised women remains unclear, though most guidelines suggest shorter intervals due to the higher incidence of cervical dysplasia in this population [36].

AIDs can complicate lymphoid and myeloid malignancies, with CLL and NHL being among the most common lymphoid proliferative diseases associated with autoimmunity [37]. Research by Herishanu et al. [38] highlights the critical role of the tumor microenvironment (TME) in CLL cell survival and proliferation. The roles of IL-17 and IL-23 in AIDs and tumor growth have been established [39, 40] and their involvement in apoptosis has also been demonstrated [41]. Autoimmune cytopenias are common in CLL and IL-17 and IL-23 are thought to jointly contribute to CLL pathogenesis. Our study confirms that MS increases the risk of several cancers: small intestine cancer (meta-*p*=0.014; OR = 2.745; 95% CI: 1.222–6.165), cervical cancer (meta-*p*=0.020; OR = 3.750; 95% CI: 1.231–11.425), and CLL (meta-*p*=0.046; OR = 1.771; 95% CI: 1.011–3.101). This suggests that MS-related autoimmune conditions may be associated with multisite cancer risk, emphasizing the need for thorough cancer screening in these patients.

CD and SLE are common autoimmune disorders. CD is characterized by discontinuous skip lesions affecting any part of the gastrointestinal tract from the mouth to the anus, often presenting as transmural inflammation with granulomas on biopsy [42]. Biancone et al. [43] studied 21,953 CD patients, identifying 99 with cancer, and found an increased cancer incidence compared to UC (*p*=0.042), suggesting that penetrating CD is a cancer risk factor. A study from Denmark including 13,756 CD patients found high risks of small intestine cancer and hepatobiliary malignancies, with stronger associations for blood system malignancies, smoking-related cancers, and melanoma [44]. The Florence inflammatory bowel disease study, which included 231 CD patients, found increased overall mortality and higher mortality from any malignancy, particularly primary respiratory and brain malignancies [45]. Our study shows a pathogenic causal effect of CD on esophagus cancer (meta-*p*=0.0014; OR = 1.064; 95% CI: 1.024–1.106), providing data for future research.

SLE is a multisystem chronic AID with a relapsing and remitting course, predominantly affecting women of childbearing age at a ratio of 9:1 [46]. Environmental and genetic factors interact to trigger immune responses, resulting in overproduction of pathogenic autoantibodies and cytokine dysregulation, leading to tissue and organ damage. SLE is characterized by the presence of antinuclear and anticytoplasmic antibodies [47]. Various risk factors, such as immunosuppressive therapy, clinical factors, viral agents, genetics, and others, may influence the link between SLE and cancer. Understanding the mechanisms of specific cancers in SLE may aid in better prevention and management of cancer incidence [48–50]. Analysis by Lu et al. [51] indicated that SLE patients typically present with myeloid malignancies, with myelodysplastic syndromes (MDSs) and AML being most common [51–53]. Our study reveals that SLE increases the risk of eye and annexa cancers (meta-*p*=0.044; OR = 6707.672; 95% CI: 1.259–35742719.800), while reducing the risk of AML (meta-*p*=0.040; OR = 0.775; 95% CI: 0.607–0.989). In summary, SLE has a pathogenic causal effect on eye and annexa tumors, while providing a protective effect against AML, with specific disease mechanisms warranting further investigation.

This study utilized a dual-database approach and included external validation, enhancing the reliability of the findings. The large dataset involved in this research covers a wide range of phenotypes, contributing to its comprehensiveness. Additionally, the use of SNPs as genetic instrumental variables ensures genetic randomness, minimizing certain sources of bias. However, the study has notable limitations. First, the population sampled is not fully representative, primarily comprising individuals of European descent. Second, due to the stringent quality control measures applied in MR, some phenotypes were excluded, and their causal effects on outcome factors require further investigation. Last, the study did not account for potential confounders such as age and gender. Future research should design more robust clinical trials to further validate these results.

## 5. Conclusions

In summary, our analysis reveals causal relationships between various AIDs and cancer at the genetic level. Notably, the finding that MG increases the risk of oral cavity cancer addresses a significant gap in existing research. This discovery not only provides a valuable reference for future observational studies but also offers a unique perspective on early cancer screening and prevention for patients with AIDs.

## Figures and Tables

**Table 1:** The main meta resluts of MR analysis of autoimmune diseases to the risk of pan-cancers.

Exposure	Outcome	Random_*p*-value	Random_OR	Random_95lci	Random_95uci	*Q*	df.Q	*p*-value.Q	I2
Type I diabetes	Esophagus	0.001	1.064	1.024	1.106	0.808	1	0.369	0
Myasthenia gravis	Oral cavity cancer	0.004	53.343	3.509	810.950	0.977	1	0.323	0
Multiple sclerosis	Small intestine	0.014	2.745	1.222	6.165	0.554	1	0.457	0
Multiple sclerosis	Uterine cervix	0.020	3.750	1.231	11.425	0.012	1	0.914	0
Systemic lupus erythematosus	Acute myelocytic leukemia	0.040	0.775	0.608	0.989	0.041	1	0.839	0
Systemic lupus erythematosus	Eye and annexa	0.044	6707.672	1.259	35742719.805	0.888	1	0.346	0
Multiple sclerosis	Chronic lymphocytic leukemia	0.046	1.771	1.011	3.101	0.249	1	0.618	0

## Data Availability

All data in this study were sourced from GWAS data at the summary level of UK Biobank processed by MRC-IEU. The complete data was derived from https://gwas.mrcieu.ac.uk/.

## Nomenclature

AIDs: Autoimmune diseases
MR: Mendelian randomization
SLE: Systemic lupus erythematosus
MS: Multiple sclerosis
CD: Crohn's disease
UC: Ulcerative colitis
MG: Myasthenia gravis
RA: Rheumatoid arthritis
SNPs: Single nucleotide polymorphisms
TWAS: Transcriptome-wide association study
MAFs: Minor allele frequencies
IVW: Inverse-variance weighted
OR: Odds ratio
LOO: Leave-one-out
IQR: Interquartile range
CLL: Lymphocytic leukemia
NHL: Non-Hodgkin lymphoma
TME: Tumor microenvironment
AML: Acute myeloid leukemia.

## Supporting Information

Supporting Information 1 Table S1: The IVs SNPs involved in autoimmune diseases (data from FinnGen database) and pan-cancers. Table S2: The IVs SNPs involved in autoimmune diseases (data from other databases) and pan-cancers of UKB database. Table S3: The single SNP's effect estimated in autoimmune diseases (data from FinnGen database) and pan-cancers. Table S4: The single SNP's effect estimated in autoimmune diseases (data from other databases) and pan-cancers of UKB database.

Supporting Information 2 Table S1: The results of MR analysis between autoimmune diseases (data from FinnGen database). Table S2: The results of MR analysis between autoimmune diseases (data from other databases) and pan-cancers of UKB database.

Supporting Information 3 The meta resluts of MR analysis of cystatins to the risk of pan-cancers.
